# Oxidative Dry Reforming of Methane in a Reactor with a Porous Membrane Catalyst

**DOI:** 10.3390/membranes16040145

**Published:** 2026-04-11

**Authors:** Mikhail Tarasenko, Andrey Makarov, Mark Neshin, Valery Skudin, Roman Kozlovskiy, Maria Myachina, Natalia Gavrilova

**Affiliations:** 1 Department of Chemical Technology of Natural Energy Carriers and Carbon Materials, Faculty of Petrochemistry and Polymer Materials, D. Mendeleev University of Chemical Technology of Russia, Miusskaya sq., 9, 125047 Moscow, Russia; tarasenko.m.a@muctr.ru (M.T.); makarov.a.s@muctr.ru (A.M.); 210957@muctr.ru (M.N.); skudin.v.v@muctr.ru (V.S.); 2 Department of Chemical Technology of Basic and Petrochemical Synthesis, Faculty of Petroleum Chemistry and Polymers, D. Mendeleev University of Chemical Technology of Russia, Miusskaya sq., 9, 125047 Moscow, Russia; kozlovskii.r.a@muctr.ru; 3 Department of Colloid Chemistry, Faculty of Natural Science, D. Mendeleev University of Chemical Technology of Russia, Miusskaya sq., 9, 125047 Moscow, Russia; miachina.m.a@muctr.ru

**Keywords:** membrane reactor, dry reforming of methane and oxygen dry reforming of methane, distributor mode, activated mass transfer, autothermal reforming of methane, conjugation of the energy released in the reaction with mass transfer

## Abstract

Oxidative dry reforming of methane (ODRM) in a membrane reactor can become the basis for creating an energy-efficient process for converting greenhouse gases into a sought-after chemical raw material for gas chemistry. The process was carried out in a distribution mode in a reactor with a membrane porous catalyst (MPC) at a temperature of 850 °C. The reagents CH_4_ and CO_2_ were supplied to the MPC through a volume of retentate, and O_2_ mixed with N_2_ through a volume of permeate. The mixture of reaction products was removed from the shell side. In the experiment, the effect of the O_2_/CO_2_ ratio on the conversion of CH_4_, CO_2_ and O_2_, as well as on the thermal effect of the process, was established. When oxygen enters the reactor during dry reforming of methane (DRM), the temperature inversion in the volumes of retentate and permeate occurs, as well as a decrease in electricity consumption in the resistor furnace. The observed effects of the ODRM process in MPC were interpreted using the hypothesis of active mass transfer occurring in pore channels. It is assumed that part of the carbon deposits in MPC will be gasified by oxygen.

## 1. Introduction

Currently in the field of hydrogen energy those areas that are associated with the implementation of the concept of decentralized hydrogen production directly at the place of consumption are actively developing. Hydrogen for fuel cells can be produced from various hydrogen-containing raw materials (saturated hydrocarbons, monatomic alcohols, ammonia) [1,2,3]. To produce such hydrogen it is necessary to use compact and efficient installations [4,5].

Large-scale processes have achieved high efficiency due to the scaling and optimization of chemical technology systems. At the same time, the development of compact plants for the on-site production of high-value-added products is challenging. The intensification of these processes is achieved by creating catalytic membrane or microreactor systems.

Dry reforming of methane (DRM), in which carbon dioxide is used as an oxidizer, is considered a promising and in demand process. Despite the fact that DRM is characterized by a large endothermic effect, combining this process with exothermic stages involving oxygen, could make it the basis for creating small-scale and environmentally attractive installations for producing synthesis gas and its products. The use of modern membrane and/or adsorption plants for the production of concentrated oxygen for combined processing of natural gas [6] will expand the scope of methane applications.

A common disadvantage of methane conversion processes with water vapor and carbon dioxide, with the exception of processes using oxygen, is the need for significant compensation for heat loss at the endothermic stages. The use of oxygen in these processes suggests the possibility of carrying out oxidative methane conversion in thermoneutral or autothermal modes [7], which should have a positive effect on the economic performance of the conversion.

Oxidative dry reforming of methane (ODRM) is a combined process in which an endothermic reaction is conjugated with an exothermic one, which should help reduce the energy costs of its implementation. A similar variant of the combined conversion process was proposed in [8,9,10], which was called tri-reforming. It was proposed to use water vapor, carbon dioxide and oxygen simultaneously as oxidizing agents. Later in [11,12] catalysts for such processes were developed.

In a traditional fixed-bed catalyst reactor, problems arise in the autothermal process due to uneven heating of the catalytic layer [13]. In such a reactor, it is difficult to ensure effective conjugation of the exothermic and endothermic stages. Perhaps one of the possible solutions could be the use of membrane reactors. In this context, the concept of using membrane catalysis becomes particularly relevant. Under certain conditions, the use of membrane catalysts can ensure energy-efficient coupling of reactions with mass transfer processes [14,15], preventing overheating and supercooling of the catalysts. The term “coupling” (or better “conjugation”) should be understood as the mutual influence on each other and the mutual dependence of chemical reaction and mass transfer on catalytic membrane catalysts.

An analysis of the publications shows that the greatest attention of researchers has been paid to reactors in which hydrogen-selective membranes are combined with a fixed bed or fluidized bed of a traditional catalyst in one housing [16,17,18]. The main problems of reactors with traditional catalysts are kinetic and thermodynamic limitations. In the processes of steam and dry reforming of methane on a stationary conventional catalyst, these limitations significantly reduce the efficiency of the catalytic reactor [6].

A successful example of experimental overcoming of these limitations is research in membrane reactors with metal non-porous membranes made of palladium, silver and their alloys. It is based on the results obtained by such membranes that the main features and advantages of membrane catalysts and membrane catalysis, respectively, were formulated by academician V.M. Gryaznov [14]. These are the phenomena of selective mass transfer and coupling in complex chemical reactions. The results of these studies were the following features that were observed in reactors with metal membranes. The coupling of chemical reactions involving selective mass transfer of hydrogen was accompanied by an intensification of chemical reactions, a change in chemical equilibrium, and an effective transfer of free energy from dehydrogenation reactions to hydrogenation reactions. Moreover, the authors of these studies identified subspecies of coupling—energy, thermodynamic and kinetic. In the works of this school, it was noted that such coupling is impossible on porous catalytic membranes. This conclusion probably arose due to the fact that these phenomena have always been reproduced only when using dense metal membranes. Of the above-mentioned features, the main parameter in the vast majority of studies suggested that it is the selective mass transfer that provides the advantages of a membrane reactor. The high purity of the hydrogen migrating through the palladium membrane was much easier to determine than the transfer of the free energy of a chemical reaction to the rate of hydrogen migration or to another reaction. Perhaps this is one of the reasons for the decrease in attention to membrane catalysis in the last decade.

Porous membranes have high permeability compared to dense membranes, but their selectivity was far from ideal. The ability to provide coupling of chemical reactions and mass transfer in a reactor with a fixed catalyst layer in the presence of porous membranes and membrane catalysts has not been discovered by the researchers. The idea of the possibility of conjugation on a membrane catalyst was not widely accepted in the scientific community at that time and remains so to this day.

However, the high permeability of porous membranes could be useful if the catalyst layer is placed on the outer or inner surfaces of tubular membranes. It can be assumed that the use of such a catalytic membrane (or porous membrane catalyst) in the mode of an intraporous membrane contactor with forced transport of reagents would overcome kinetic limitations. Reactions with kinetic limitations include reactions occurring at high temperatures, and, in particular, reactions of steam, dry, and partial reforming of methane (reactions 1–3) in Table 1. Each of these reactions is accompanied by a water gas shear reaction (4).

To establish the fact of intensification of any of these reactions, it is necessary to compare a reactor with a stationary layer of a traditional catalyst and a reactor with a porous membrane catalyst. In such a comparison, it is necessary to use catalysts (traditional and membrane) with an identical chemical composition of the active component and the same experimental conditions. In this case, it will be possible to establish, for example, the methane cracking rate constants in both reactors and draw an appropriate conclusion about mass transfer from them.

In the kinetic experiment [15], the dry methane reforming reaction on traditional (powdered) and membrane catalysts with an active component, molybdenum carbide, was chosen as a model reaction. The intensification phenomena were established, and the rate constants of direct and reverse reactions at intermediate stages were determined.

It should be noted that all methane reforming reactions include several stages, each of which is usually considered reversible. The most thermodynamically probable stage for reactions (1)–(3) is the methane cracking reaction (5) [19] in Table 1. In this reaction, two products are formed: hydrogen and carbon (the latter in the form of carbonaceous deposits).

It is generally assumed that the absence of carbon accumulation on a traditional catalyst during the methane reforming process indicates the processes of carbon deposition gasification with water vapor (6), carbon dioxide (7) and oxygen (8), respectively, for reactions (1)−(3) in Table 1. The main product of all gasification reactions is carbon monoxide, while in the steam gasification reaction (6), hydrogen is also formed. These reactions (6)–(8) in Table 1 can prevent the accumulation of carbon on the surface of the catalysts.

The equilibrium between the gaseous products in all reforming reactions is established in the direct or reverse reaction shift water gas (4) in Table 1. In this case, in the reverse shear reaction of water gas, hydrogen interacts with carbon dioxide and turns into water and carbon monoxide.

To explain the observed phenomena in a kinetic experiment on a porous membrane catalyst, the hypothesis was used that thermal slip (thermal transpiration, thermal creep) is the main physical phenomenon leading to the intensification of chemical reactions and a change in chemical equilibrium. In the framework of nonequilibrium thermodynamics, the phenomenon of thermal slip is considered a phenomenon of active mass transfer, in which a substance is transferred against a chemical potential gradient. Namely, from a less heated area to a more heated one.

At the time of the formulation of the basics of membrane catalysis, the concepts of mass transfer of gases in porous media under conditions of chemical reactions differed significantly from modern ones [20]. Although the thermodynamic analysis presented in [21] was published around the same time and indicated a significant increase in kinetic constants by several orders of magnitude. An analysis of the results of a kinetic experiment in dry methane reforming has shown the possibility of coupling and intensification of chemical transformation processes in pores corresponding to Knudsen diffusion, when active mass transfer occurs in them. In the last decade, kinetic studies of DRM have been conducted in the main operating modes of a reactor with a membrane porous catalyst (MPC): contactor, extractor, and distributor [15,22]. In these DRM studies, a kinetic scheme of intermediate stages was used, including reactions (I)–(IV).

Kinetic scheme of DRM on a porous membrane catalyst:

(I) $$\begin{array}{c} \;\;\;\;\;\;\;\;\;\;k_{1}\\ {CH}_{4}\;↔\\ \;\;\;\;\;\;\;\;\;\;\;k_{2} \end{array}\;C+{2H}_{2}$$

(II) $$\begin{array}{c} \;\;\;\;\;\;\;\;\;\;\;\;\;\;\;\;\;\;\;k_{3}\\ C+{CO}_{2}\;↔\\ \;\;\;\;\;\;\;\;\;\;\;\;\;\;\;\;\;\;\;k_{4} \end{array}\;2CO$$

(III) $$\begin{array}{c} \;\;\;\;\;\;\;\;\;\;\;\;\;\;\;\;\;\;\;\;\;k_{5}\\ {CO}_{2}+H_{2}\;↔\\ \;\;\;\;\;\;\;\;\;\;\;\;\;\;\;\;\;\;\;\;\;k_{6} \end{array}\;CO+H_{2}O$$

(IV) $$\begin{array}{c} \;\;\;\;\;\;\;\;\;\;\;\;\;\;\;\;\;\;{\;\;k}_{7}\\ C+H_{2}O↔\\ \;\;\;\;\;\;\;\;\;\;\;\;\;\;\;\;\;\;\;\;k_{8} \end{array}\;CO+H_{2}$$

In order to establish the expected conjugation of exothermic and endothermic processes, it is necessary to know the complete composition of the products of ODRM in a membrane reactor, the corresponding thermal effect and changes in these parameters over time on stream. Only after that will it be possible to make changes or additions to the kinetic scheme of this process and begin the kinetic experimental study.

The coupling of dry reforming and partial oxidation in this work was dictated, not least, by the fact that the studies carried out earlier would make it possible to use previously developed methods of experimental approach, processing and analysis of the results of a kinetic experiment on a membrane catalyst in DRM.

The purpose of this work is to carry out dry reforming of methane with mixtures of oxygen and carbon dioxide on porous membrane catalysts and to hypothesize the reaction of these DRM products with oxygen.

## 2. Materials and Methods

### 2.1. Characteristics of Membrane Catalyst

Asymmetric tubular membranes (Genos LLC, Moscow, Russia) made of α-Al_2_O_3_ with an outer diameter of 8 mm and a wall thickness of 1.1 mm, with a microfiltration layer of α-Al_2_O_3_ particles with a size of 2 μm, were used as a substrate for creating membrane catalysts. A CVD reactor (laboratory level) with “cold” walls and a circulation circuit for the combined-gas composition (Ar and carbonyl vapor) was used to form a catalytic layer on the surface of the substrates. The catalytic layer of the binary composition MoO_2_-WO_2_/α-Al_2_O_3_ was obtained in several stages: first, a barrier layer of tungsten dioxide (1 wt.%) was formed on the initial substrate by chemical deposition from the gas phase (CVD) at a substrate surface temperature of 350 °C, the sublimation temperature of tungsten hexacarbonyl (W(CO_6_) 80 °C and at atmospheric pressure in the reactor. The applied barrier layer was calcined in an inert argon atmosphere at a temperature of 400 °C for 1 h. Then, a catalytic layer of binary composition was formed in a stoichiometric ratio of 50:50 (MoO_2_:WO_2_) from a mixture of molybdenum and tungsten carbonyl vapors at a substrate surface temperature of 250 °C and the same sublimation temperature of the carbonyl mixture. In order to ensure an even distribution of the catalytic layer along the length of the membrane, the deposition process was repeated an even number of times, while changing the position of the membrane. The total formation time of the catalytic layer was 12 h. The uniformity of the distribution of the catalytic layer over the surface of the substrate was estimated by the distribution of electrical resistance along the length of the sample. MC activation was performed in a membrane reactor using temperature-programmable reduction (TPR) in the flow mode of the membrane reactor (contactor).

The following were used as initial volatile compounds in the CVD process: molybdenum hexacarbonyl (Mo(CO)_6_, TS 6-02-968-74, Mo(CO)_6_ content 98.5 wt.%, ACROS organics, Geel, Belgium) and tungsten hexacarbonyl (W(CO)_6_, TS 6-02-728-78, ACROS organics, Geel, Belgium).

X-ray diffraction (XRD) patterns were recorded at room temperature over the scanning range (2θ) of 20–90° with a step 0.020° and scan step of 5°min^−1^ using a Rigaku D/MAX 2500 diffractometer (Rigaku Corporation, Tokyo, Japane) with CuKα radiation. Accelerating voltage was 50 kV, cathode filament current 250 mA (total X-ray tube power—12.5 kW). The phases present in the samples were identified using JCPDS Powder Diffraction File data.

The samples’ morphology was studied using a Jeol JSM 6510 scanning electron microscope (Jeol Ltd., Tokyo, Japane) with an EDX + SSD X-MAX X-ray microanalysis system.

The specific surface area, pore volume were calculated on the basis of data of N_2_ low-temperature adsorption on a Gemini VII (Micromeritics, Norcross, GA, USA) surface area and porosity analyzer. The specific surface area of the samples was calculated using the BET method. The total specific pore volume was determined at a maximum relative pressure of 0.995.

### 2.2. Methodology of Temperature-Programmed Reduction of Membrane Catalyst

The reactor with a membrane catalyst was heated using a resistance furnace connected to a temperature sensor at a rate of 1200 deg/h to 400 °C in a flow of inert gas—high purity nitrogen (50 mL/min). Temperatures in the retentate (R) and permeate (P) areas were monitored simultaneously. Upon reaching 400 °C, hydrogen was added to the gas flow, forming a gas mixture of H_2_:N_2_ = 80:20 vol.%, while the total volumetric flow rate was maintained. The heating rate was reduced to 200 °C/h and maintained until the final reduction temperature was reached. Upon reaching a temperature of 700 °C, the H_2_ supply was stopped, while the nitrogen supply was maintained. Simultaneously, a mixture of CH_4_:CO_2_ = 50:50 vol.% (in a stoichiometric ratio) was fed to the retentate.

### 2.3. Experimental Method of DRM and ODRM

The membrane reactor is made of stainless steel (inner diameter = 28 mm; length = 350 mm), in which a tubular catalytic membrane (Genos LLC, Moscow, Russia) is placed on the holder (outer diameter = 8 mm; working surface length = 31 mm).

A tubular membrane catalyst with a metal oxide layer(s) on its outer surface was sealed on one side with DoneDeal DD6785 high-temperature ceramic sealant (Done Deal Adhesives Lab, Inc., Hudson, MA, USA) and secured in a holder on the other side of the membrane reactor. After installing the membrane catalyst, the reactor was leak-tested by applying nitrogen at a pressure of 0.2 MPa. The absence of a pressure drop in the reactor confirmed its gas tightness.

**Figure 1:**
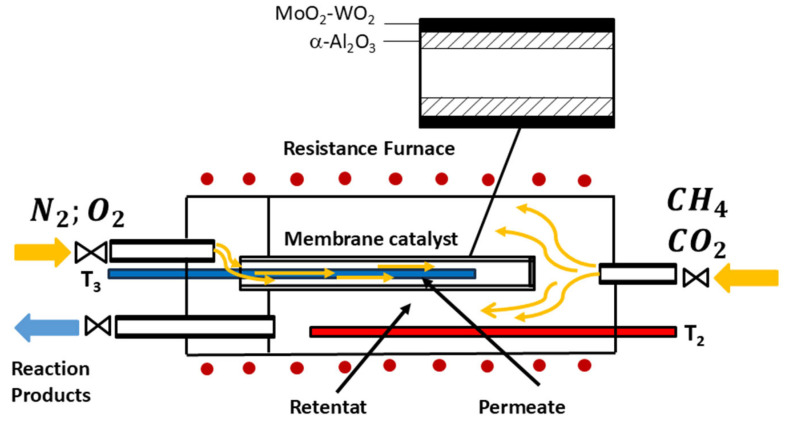
Conceptual scheme of the separate reagent supply in the distributor mode in a reactor with a porous membrane catalyst.

The study of the DRM and ODRM was conducted by sequentially switching between modes during a continuous experiment. In the reactor, a tubular membrane catalyst with a catalytic layer applied to the outer surface of the membrane (Figure 1) divides the reaction space into two parts—the retentate and the permeate, which can communicate exclusively through pore channels. Distributor mode was established by separately feeding the reaction mixture components as follows. The reactant mixture (CH_4_ and CO_2_) for DRM was fed to the retentate (shell side)—while nitrogen or its mixture with oxygen was fed to the permeate (tube side). Distributor mode ensures uniform distribution of one of the reaction mixture components (specifically, oxygen) throughout the membrane catalyst, preventing localized overheating in exothermic reactions or undercooling in endothermic reactions.

Initially, the reaction was run in flow-through contactor mode, feeding a mixture of carbon dioxide and methane into the annular space of the reactor (the retentate volume or shell side) on the catalyst layer. Once the temperature reached 850 °C, a gas mixture of CH_4_:CO_2_ = 1:1 reagents with a volumetric flow rate of 50 mL/min was fed into the permeate volume (or tube side) and stationary conditions in DRM were established. The composition of the reaction mixture exiting the reactor was determined to monitor the catalytic activity of the catalyst used throughout the experiment.

The membrane reactor then was switched to distributor mode by feeding nitrogen into the permeate volume for safety reasons. Continuously feeding nitrogen into the reactor’s internal volume maintains the hydrodynamic pattern of the distributor mode, regardless of the presence of oxygen in the mixture, and allows for monitoring of the change in the volume of the reaction medium resulting from methane conversion.

After steady-state conditions were established in distributor mode, the DRM was switched to the ODRM process by adding oxygen to the nitrogen feed. This created a gas mixture of N_2_ and O_2_ at permeate inlet with a total flow rate of 92 mL/min (N_2_—23.8 vol.% and O_2_—20 vol.%) and CH_4_ and CO_2_ at retentate inlet with a total flow rate 118 mL/min (CH_4_—38.1 vol.% and CO_2_—18.1 vol.%). The reaction mixture formed during the DRM or ODRM was removed from the retentate. A portion of that flow was periodically sampled for analysis to determine its composition by chromatography. Both incoming reactant streams entered the reactor in a counterflow mode. The reactor temperature was maintained constant (850 °C) throughout the experiment. The composition of the mixtures was determined after the concentrations stabilized.

**Figure 2:**
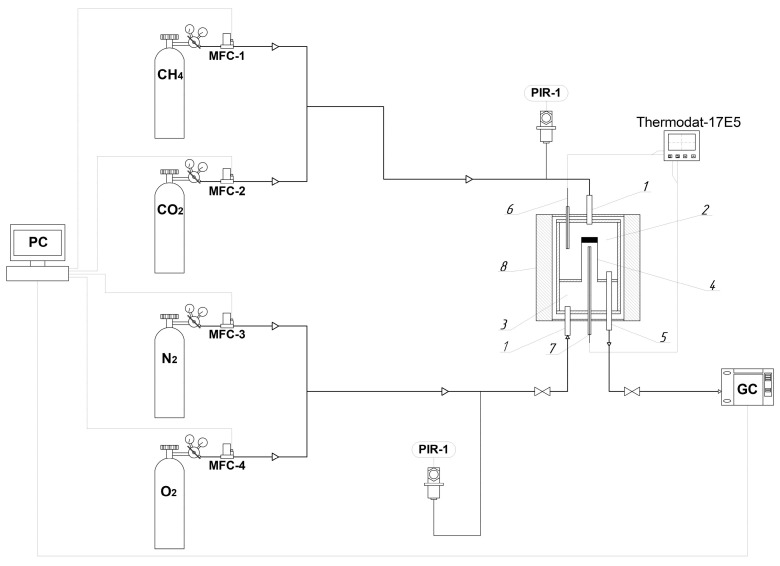
Scheme of the reactor with a membrane catalyst: 1—feedstock inlet; 2—retentate section; 3—permeate section; 4—membrane catalyst; 5—reaction mixture outlet; 6, 7—thermocouples; 8—resistance furnace.

The composition of the flows entering the reactor was set and maintained constant using mass flow meters controllers (in the diagram—MFC-1–4) (Bronkhorst High-Tech B.V., Ruurlo, The Netherlands) in Figure 2. The composition of the gas mixture at the reactor inlet in the DRM was as follows: CH_4_—38.1 vol.%; CO_2_—38.1 vol.%; N_2_—23.8 vol.%, and in ODRM: CH_4_—38.1 vol.%; CO_2_—18.1 vol.%; O_2_—20 vol.%; N_2_—23.8 vol.%. The temperature in the retentate and permeate was controlled by thermocouples (pos. 6 and 7). The thermal mode of the resistor furnace (pos. 8) and the power consumed by it were controlled by the Termodat—17E5 (NPP Control Systems LLC, Perm, Russia) regulator according to the readings of the thermocouple (pos. 7). During operation, the controller continuously measured the current temperature after 1.2 s and compared it with the set value. Using a PID controller, the device calculated the output power of the resistor furnaces (in percentages) at any given time. These values were transmitted via the built-in interface to the MasterSCADA software (v3.7) (MPS Soft, Moscow, Russia), which controlled the entire process. The composition of the reaction mixture formed in the DRM and ODRM was determined using a Crystallux-4000 M chromatograph (NPF Meta-Chrom LLC, Yoshkar-Ola, Russia). All monitored and control parameters were displayed and set from a computer (PC).

Each of these modes is characterized by different power consumption, which can be estimated by the value recorded by the temperature controller and representing the percentage of the maximum possible power consumption of the furnace heating the reactor. In our experiment, the zero-power consumption state was defined as the percentage of the furnace’s maximum power before reactants were introduced into the reactor. Under carbon dioxide reforming conditions, the percentage of the furnace’s maximum power should increase; in oxygen–carbon dioxide reforming of methane, it may remain the same as before reactants were introduced (thermoneutral process) or decrease (autothermal process).

Reagent conversions were calculated using the following formulas:

(1) $$X_{CH_{4}}\left(\%\right)=\frac{\left(F_{CH_{4},in}-F_{CH_{4},out}\right)}{F_{CH_{4},in}}\times 100$$

(2) $$X_{CO_{2}}\left(\%\right)=\frac{\left(F_{CO_{2},in}-F_{CO_{2},out}\right)}{F_{CO_{2},in}}\times 100$$

(3) $$X_{O_{2}}\left(\%\right)=\frac{\left(F_{O_{2},in}-F_{O_{2},out}\right)}{F_{O_{2},in}}\times 100$$

The consumption of the components at the outlet was determined indirectly by nitrogen, which was not consumed during the reaction and was the internal standard:

(4) $$F_{total,out}=\frac{F_{N_{2},in}}{y_{N_{2},out}}$$

(5) $$F_{j,out}=y_{j,out}\cdot F_{total,out}$$

where X(CH_4_), X(CO_2_), X(O_2_) are the conversions of CH_4_, CO_2_, and O_2_, respectively; F_j,in_, F_j,out_ are the volume flow of components j at the reactor inlet and outlet, ml/min (under normal conditions); y_j,out_ is the volume fraction of the component at the reactor outlet.

## 3. Results

### 3.1. Characteristics of the Membrane Catalyst

**Figure 3:**
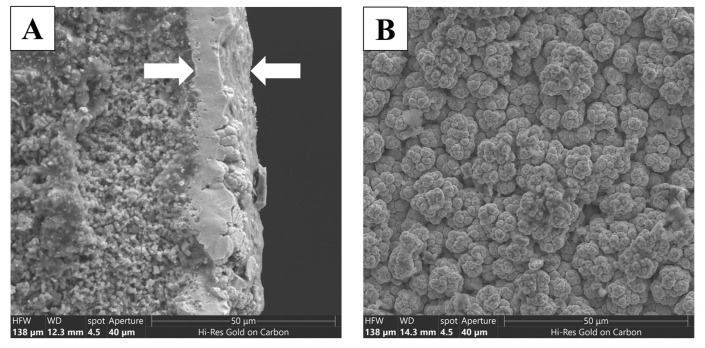
Micrograph of the cross section (**A**) and the outer surface (**B**) of the membrane catalyst.

Figure 3A shows an image of a cross-section of a membrane catalyst, which shows that the catalytic layer is located on the outer surface of the substrate and its thickness is approximately 12.5 microns. The microfiltration layer and the partially supporting layer of the α-Al_2_O_3_ substrate are also visible in this image. Figure 3B shows the outer surface of the membrane catalyst, on which one can observe the structures of spheroids, which are characteristic of the formation of a layer by the CVD method. It can be seen that there are no major defects in this layer. According to the EDX results, the metal content in the catalytic layer was 63 wt% Mo and 37 wt% W.

The main characteristics of the synthesized membrane catalyst are shown in Table 2.

### 3.2. Conversion of Methane, Carbon Dioxide and Oxygen in DRM and ODRM Processes

In the experiments presented below, the conversion in the processes of DRM and ODRM was determined.

**Figure 4:**
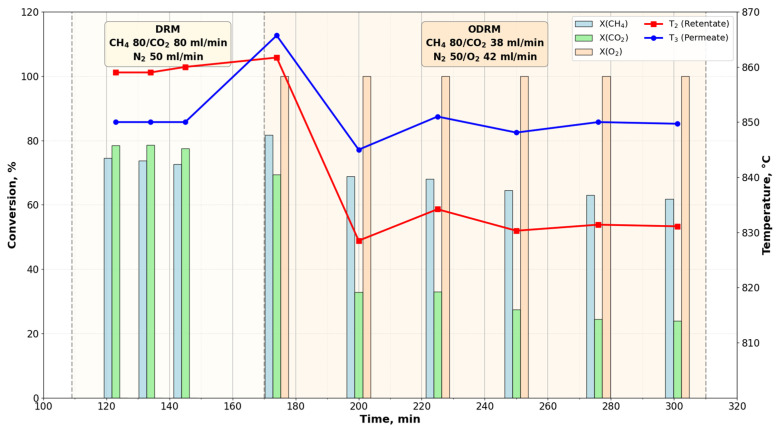
Dependence of the conversion of methane, carbon dioxide, and oxygen on time in DRM and ODRM processes on a porous membrane catalyst (the standard deviation for DRM and ODRM is 2.1 vol.% and 6.5 vol.%, respectively).

Figure 4 shows the conversion values of methane, dioxide, and oxygen changes over time, and the temperature changes in both parts of the reactor (in retentate and permeate).

In the absence of oxygen supply to the reactor, the conversion of methane and carbon dioxide (DRM) reached moderate values (73–75% for methane and 78% for carbon dioxide, respectively). Under these conditions the temperature of the reaction mixture in the retentate (860 °C) was approximately 9–10° higher than in the permeate (850 °C). This temperature difference is a consequence of endothermic reactions occurring within the pores of the membrane catalyst.

After oxygen was supplied and the concentration of carbon dioxide at the reactor inlet was reduced, the conversion of methane decreased, from 82% to 62%, and carbon dioxide, from 69% to 24%. The conversion of oxygen during the ODRM turned out to be complete (100%) and remained so throughout the experiment. Figure 4 shows that the conversion of methane under DRM conditions exceeded the conversion under the conditions of the combined process (ODRM). At the same time, the conversion of carbon dioxide decreased by almost 3 times. The temperature in the permeate remained at the same level—850 °C, and in the retentate it decreased from 860 °C to 831 °C.

### 3.3. Heat Consumption in the DRM and ODRM Processes

**Figure 5:**
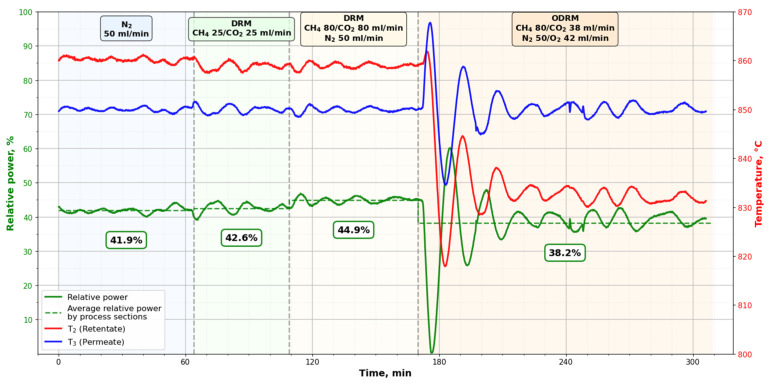
Dependence of the relative power of the installation, temperatures in the reactor volumes on volume flow rate, composition of gas mixtures and time.

Figure 5 shows that the addition of oxygen led to the process being accompanied by heat generation, a change in the temperature of the reaction mixture in the volume of retentate, and a decrease in energy consumed during the ODRM process. The change that is difficult to explain in this graph is a decrease in temperature in the retentate, the volume of the reaction space into which heat is supplied directly from the heating furnace. If we consider the DRM process involving oxygen, then judging by the temperature differences at the boundaries of the retentate, it can be assumed that heat from the furnace and from the tube side should have been transferred to the shell side, since the temperature in it turned out to be the lowest. In DRM without oxygen, the heat from the furnace was sufficient to achieve higher temperatures in the retentate.

A change in the process parameters was observed throughout the experiment: first in the absence of chemical transformations (when only nitrogen was supplied to the reactor), then in the DRM mode with different compositions and flow rates of the reagent mixture at the reactor inlet, and then in the ODRM mode. The supply of oxygen causes an increase in the temperature in the permeate and is also accompanied by an increase in the amplitude of fluctuations of both temperatures and power consumption. In the figure, for each case of gas mixtures being supplied to the reactor, it can be seen that the consumption level for all studied reagent mixtures varied. For each of the four sections, the graph shows a horizontal dotted line, which corresponds to the average values of energy consumption as a percentage of the maximum power of the resistor furnace.

In particular, the average power consumption with nitrogen alone was 41.9%. This is the heat output of the heater, which is used to compensate for heat losses in the surrounding space. When nitrogen was replaced by a mixture of carbon dioxide and methane in an equimolar ratio and while maintaining volumetric flow rate, energy consumption increased to 42.6%. When nitrogen was added to the reagents and the flow rate of the mixture increased from 50 mL/min to 210 mL/min, energy consumption increased to 44.9%. The observed increase in energy consumption is due to the endothermic effect of the carbon dioxide conversion reaction and methane. Compared with the losses to the surrounding space, this increase is insignificant and can be explained by the small size of the MPC (*l* = 46 mm; the length of the cylindrical working surface is 31 mm) and the relatively low volume consumption of the reagent mixture. The high temperature of the process (850 °C), the large heat exchange surface and the mass of the metal reactor cause high losses to the surrounding space. However, despite this, the results obtained allow us to assess the contribution of the exothermic partial oxidation reaction to the endothermic process of carbon dioxide conversion of methane.

Oxygen supply to this reaction mixture was accompanied by a reduction in energy consumption to 38.2%. Although the difference between the maximum and minimum consumption in our experiment is not large, it allows us to compare the costs of endo- and exo-processes in DRM and ODRM.

The results obtained confirm that, indeed, the conversion of methane and carbon dioxide is an endothermic process. DRM energy consumption increases compared to the nitrogen supply mode in the reactor. As the volume flow of the reaction mixture increases (from 50 mL/min to 210 mL/min), the heat costs of the DRM process also increase. When oxygen was added to the reagent mixture, energy consumption decreased, and its amount turned out to be sufficient to compensate for the endothermic effect in the DRM and partially compensate for losses to the environment.

### 3.4. Time Dependence of Reagent Concentrations in DRM and ODRM Processes

**Figure 6:**
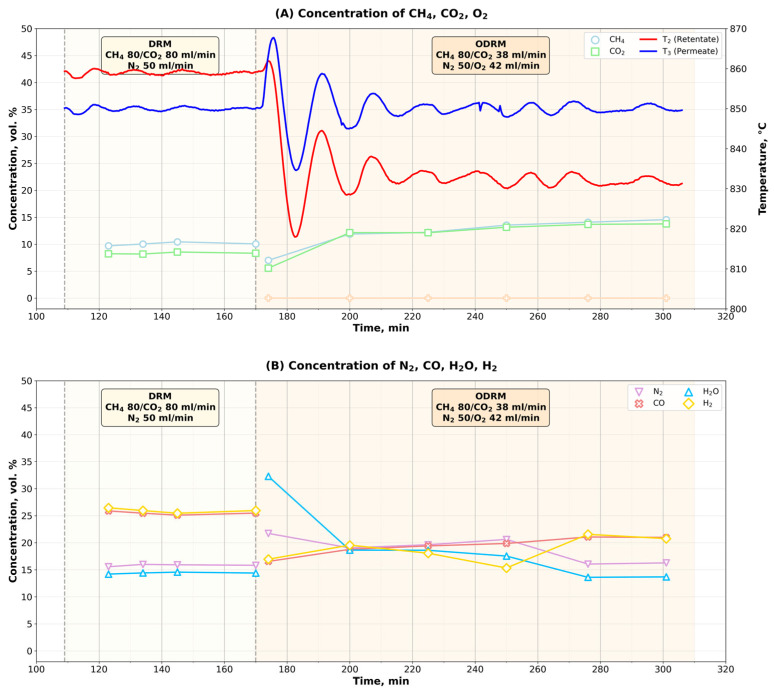
Dependence of concentrations of reagents (**A**) and products (**B**) in DRM and ODRM processes on time.

A temperature inversion was observed during the transition from the DRM to the ODRM. In Figure 6, three periods of the experiment can be distinguished. At the beginning of the experiment, the DRM process was initiated in the reactor. After the stationary process was established, oxygen was introduced into the reaction mixture. This is the beginning of the second period in which the DRM process is transformed into ODRM. Since the same catalyst was used in both processes, the addition of a new component (O_2_) is probably due to the adaptation of the catalyst with it. And in the third period, a stationary ODRM process was established.

Figure 6A,B show the change in temperature in the retentate and permeate volumes, as well as the change in the concentrations of the reactants (CH_4_, CO_2_, O_2_) and reaction products (CO, H_2_O, H_2_), respectively. The reactor inlet compositions during the DRM process differed quantitatively from those in the ODRM in terms of oxygen-containing reagents (CO_2_—18.1 vol.% and O_2_—20 vol.%).

During the DRM process, CO_2_ and CH_4_ concentrations decreased from their initial values (18.1 vol.% and 38.1 vol.%, respectively) to approximately 8–10 vol.%. At the same time, their concentrations became closer to each other, but they were higher in ODRM than in DRM. As a result of the carbon formed during the cracking of methane (5) and its further interaction with oxygen, the concentration of carbon monoxide in the reaction mixture increases. It can be assumed that the equilibrium in reaction (7) will change and its direction will be reversed. This reaction will become another carbon source in the ODRM and will lead to an increase in the concentrations of CH_4_ and CO_2_ in the reaction mixture. The concentration of (inert) nitrogen in the reaction mixture also decreased, indicating that the volume of the reaction mixture increased due to the formation of products (CO, H_2_, and H_2_O) in the DRM reaction. This is quite consistent with the stoichiometry of the reaction of DRM.

As can be seen in Figure 6B, the concentration of water vapor in the reaction mixture increased in the beginning of the ODRM process, which can only be formed when CO_2_ reacts with H_2_ in reaction (4). As constant conditions were reached, the water vapor concentration decreased to the values observed during the DRM process.

The concentrations of target products (CO and H_2_) in the ODRM process after oxygen addition also decreased compared to the concentrations in the DRM process. However, they remained equal in the ODRM process.

When oxygen is supplied to the reaction mixture, the concentration of carbon oxides must first increase or the ratio between the oxides must change. It is likely that the interaction of carbon dioxide with hydrogen led to an increase in the concentration of water vapor in the reverse reaction of the reverse water–gas shift (4). This reaction is homogeneous, quickly reaching equilibrium and occurring under conditions in which the gas mixture is in a state of continuity.

### 3.5. Temperature Inversion in the DRM and ODRM Processes

During the transition from the DRM process to the ODRM process, an unusual temperature change was observed in the volumes of the reactor with the MPC. When oxygen was supplied to the permeate, the temperature in the shell side decreased by almost 30° and was 20° lower than the temperature in the permeate. The temperature value in the tube side remained at the same level, since the furnace power was controlled by a thermocouple located in this reactor volume. When the oxygen supply to the permeate was stopped, the temperature in the retentate returned to its previous value. The same changes occurred when a control thermocouple was placed in the retentate. The temperature in it turned out to be lower than the temperature in the permeate.

The temperature inversion was reproduced regardless of the position of the control thermocouple—in permeate or in retentate. We were unable to detect this phenomenon—temperature inversion—in the works of other researchers. Therefore, an explanation of the phenomenon of temperature inversion is presented below, based on the hypothesis of active mass transfer on MPC, which we applied in several previous publications (Appendix A).

## 4. Discussion

It has been established that the processes of dry and oxygen dry reforming of methane in a membrane reactor produce the same products, achieve similar degrees of methane conversion, and observe ratios of target products (H_2_/CO) close to unity. These are processes with negative (DRM) and positive (ODRM) total thermal effects and with oppositely directed temperature gradients in the pore channels along the wall thickness of the membrane catalyst. It can be noted that there is a significant decrease in carbon dioxide conversion during ODRM. Among the parameters related to the features of the DRM and ODRM processes, it should be noted that there is a decrease in energy consumption and a temperature inversion in the volumes of retentate and permeate during the transition from dry reforming to oxygen dry reforming of methane. The main exothermic and endothermic reactions are localized exclusively on the membrane catalyst. This suggests an identical mechanism of both processes and the conjugation of exothermic and endothermic reactions at the intermediate stages of ODRM.

Our earlier kinetic studies of dry methane reforming in a reactor with a membrane catalyst allowed us to form a hypothesis that explained the unusual phenomena identified in this process. These features include the intensification of mass transfer, and an order of magnitude increase in rate constants of reactions at intermediate stages of the DRM process. In addition, the appearance of a temperature gradient depending on the methane conversion in the channels of the MPC, the absence of permeability dependence on external pressure of the membranes, a change in chemical equilibrium constants, and a decrease of 100–150 °C in the temperature compared to the process on a traditional catalyst of identical composition were noted [23]. This hypothesis was based on the phenomenon of active transfer. We used this hypothesis to analyze the process of ODRM on the MPC.

**Figure 7:**
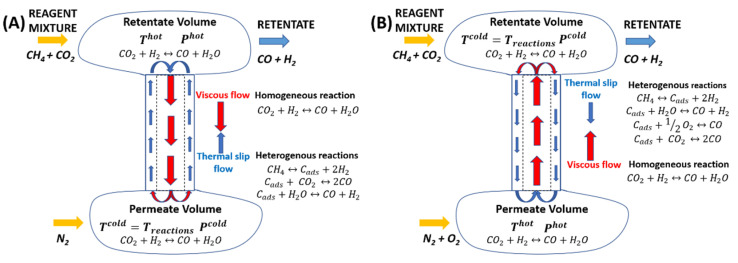
Inversion of the driving force (tangential temperature gradient) of active mass transfer in the pore channels of a membrane catalyst in processes (**A**) DRM and (**B**) ODRM in distributor mode.

Figure 7 shows the flow structures in the methane conversion processes—DRM (A) and ODRM (B) in single channel of the MPC connecting adjacent parts of the reaction space in the reactor. To form such a flow structure, the pore diameter of the catalytic layer in the MC must correspond to the conditions of Knudsen diffusion in them. The flow structures in the DRM and ODRM processes on a membrane catalyst can be obtained using numerical modeling [24], analytical solutions based on regularized equations [25] or specialized software packages (OpenFOAM) [26] or (Ansys Fluent v2018) [27]. However, it is not yet possible to link them with the results of our experiment due to the difference in conditions and tasks, because they are usually not considered a chemical reaction and are used primarily for micro electromechanical systems (MEMS). The second necessary condition is the presence of a tangential temperature gradient (along the pore channels). When these conditions are met, a specific transfer of matter from the “cold” part of the reaction space to the “heated” one occurs in the pore channels, which is called thermal slippage [20]. The thermal slip flow generates a counterflow of matter along the axis of a single pore channel. Eventually, both flow in a single channel, together with the volumes of retentate and permeate, forming a circulation circuit. In the circulation circuit, mass transfer occurs in the direction opposite to the temperature gradient and mass exchange occurs between the parts of the reaction space on both sides of the membrane catalyst. The temperature gradient and thermal slip determine the direction of circulation occurring in the single channel. The transfer of molecules in the gas layer near the wall of the pore channel, is moving due to the temperature difference (more precisely, the tangential temperature gradient), occurs under rarefied conditions and against the gradient of chemical potentials.

The thermal slip flow in one channel is indicated by the blue arrows in Figure 7A,B, and the viscous flow is indicated by red arrows.

The most thermodynamically probable of all intermediate reactions in the processes of any methane reforming is methane cracking (reaction 5 or I in the kinetic scheme) [19]. This reaction is accompanied by a strong endothermic effect and initiates all types of methane reforming. In DRM on porous membrane catalyst, when gas flows enter the pore channel from the permeable volume, methane is dissociated into carbon and hydrogen. In ODRM methane decomposition is started when gas flows entering the pore channels from the retentate volume. In addition, if the methane conversion is not more than 100%, carbon will be deposited on the channel walls along its entire length. It is at the entrance to the channels that the concentration of the components corresponds to their maximum values and, accordingly, the maximum thermal effects values of the reactions in which they participate are also reached. Moreover, the thermal effects of all heterogeneous reactions in porous channels are the sum. However, due to the rarefaction and differentiation on the molecules’ velocities, carbon gasification reactions are likely to occur in different parts of the pore channel or at different distances from its entrance. This is explained by the fact that the average speeds of molecules are determined by their molecular weight, or, more precisely, the inverse relationship between the speed and mass of molecules. Carbon gasification reactions will also begin in the channel closer to the entrance, which may involve “lighter” molecules (CH_4_ and H_2_O), while reactions involving “heavier” molecules (O_2_ and CO_2_) will occur further from the entrance. The probability of participation of oxygen in carbon gasification near the entrance to the channel will also be minimal. Closer to the outlet, the concentration of water vapor will decrease, while the concentration of oxygen will stay high. The contribution of the carbon gasification reaction with oxygen and the magnitude of its exothermic effect will increase as it approaches the exit from the pore channel into the tube side.

In the DRM and ODRM processes, respectively, the temperature in the permeate and retentate volumes is minimal, allowing them to be conventionally termed “cold” volumes of the reaction space.

In the process of DRM, this is the volume of permeate at which the methane cracking reactions (reaction I in the kinetic scheme), gasification of carbon deposits by water vapor (reaction IV) and by carbon dioxide (reaction II) begin at the entrance to the pore channel. In the ODRM process, this is the volume of retentate in which the reverse water–gas shift reaction (RWGS, reaction III) will occur, and entrance to channel to which reactions (I and IV) will begin. This fact was confirmed in our experiments by direct measurements of temperatures in retentate and permeate, which are shown in Figure 4, Figure 5 and Figure 6. These parts of the reaction volume are energy effluents in the DRM and ODRM processes.

The presence of methane at the reactor outlet means that during the reaction, one of its components (carbon) will be distributed along the entire length of the pore channel (or along the thickness of the catalytic layer on the membrane). Initially, water vapor participates in the gasification reaction, and after its exhaustion, oxygen. It is possible that carbon dioxide in the ODRM process does not react with carbon in the ODRM process at all. The control thermocouple located in both processes is located in the permeate. Therefore, during the DRM process, the lowest temperature in the reactor was observed in the retentate, while in the ODRM it was low in this volume.

As a result, during the ODRM process, the heat flow from the heater to the retentate decreased when the temperature in the tube side exceeded the set value due to an exothermic reaction with oxygen. All of the above can be considered as a mechanism by which the energy released in the chemical process was directly transferred to the heat transfer process through mass transfer in the pore channel.

## 5. Conclusions

An experimental comparison of DRM and ODRM in a reactor with the MPC was performed at a constant process temperature of 850 °C. The distribution mode of the reactor with a porous membrane catalyst was used, in which a mixture of methane and carbon dioxide was supplied to the shell side, and oxygen and nitrogen to the permeate. Nitrogen was supplied throughout the experiment, and oxygen was added to the nitrogen during the ODRM process. It was found that the supply of oxygen to the DRM reaction mixture led to a decrease in the temperature in the retentate volume below its values in the permeate (temperature inversion in the reactor) and a decrease in electricity consumption. The oxygen conversion remained complete throughout the experiment. After the oxygen supply was stopped, the temperature in the retentate returned to its original values, while the temperature in the permeate remained unchanged in both processes. In the ODRM process, the conversion of methane and, especially, carbon dioxide decreased compared to the DRM process, but the ratio between the target components of H_2_ and CO remained equal to unity. The concentration of water vapor in stationary conditions remained the same in both processes but changed significantly during the transition period after oxygen was supplied.

The temperature gradient along the length of a single pore channel is the driving force of active mass transfer due to thermal slip. It turned out that during the transition from the endothermic process of DRM to the exothermic process of ODRM, both the direction of thermal slip and the direction of circulation in a single channel of the MPC change. In DRM, gases enter the pore channels of the membrane catalyst from the tube side, where endothermic reactions of methane cracking and carbon dioxide gasification of carbonaceous deposits occur at the inlet. There was also a change in the ratio between the number of endothermic and exothermic oxidation reactions.

As a result, a low-temperature region was formed in the permeate of the DRM process—a “less heated” volume. In this case, only one reaction is localized in the retentate—the endothermic reverse reaction of the water gas shear. In the ODRM, after oxygen is supplied and the tangential temperature gradient and circulation direction are changed, the reaction mixture enters the pore channels from the shell side. The thermal effect of the endothermic reactions is compensated by the heat generated by the resistance furnace and the heat of the exothermic reactions.

The characteristics of the DRM and ODRM processes, such as the structure of gas mixture flows in pore channels, which determine the mass and heat transfer phenomena observed in them, should be attributed to conjugation processes. An analysis of the experimental results showed that the free energy of chemical reactions is conjugated with mass exchange and mass transfer. In the process of oxygen dry reforming of methane, the conjugation of endothermic and exothermic chemical reactions is added to the above-mentioned processes. From the perspective of nonequilibrium thermodynamics, the process of oxygen dry reforming can be considered as a cross-process of coupling chemical reaction and diffusion, which is characterized by efficient energy exchange [28]. In order to confirm this assumption, it is necessary to continue its study in a kinetic experiment.

## Figures and Tables

**Table 1:** Reactions of oxidative dry reforming of methane.

Reaction Number	Reaction	ΔH_298_ (kJ/mol)
(1)	CH_4_ + H_2_O ↔ CO + 3H_2_	206
(2)	CH_4_ + CO_2_ ↔ 2CO + 2H_2_	247
(3)	CH_4_ + 0.5O_2_ ↔ CO + 2H_2_	−35.6
(4)	CO + H_2_O ↔ CO_2_ + H_2_	−41
(5)	CH_4_ ↔ C + 2H_2_	74.9
(6)	C + H_2_O ↔ CO + H_2_	131.3
(7)	C + CO_2_ ↔ 2CO	172.4
(8)	C + 0.5O_2_ ↔ CO	−110

**Table 2:** The main characteristics of the synthesized membrane catalyst.

Phase Composition	Surface Area, m^2^/g	Pore Volume, sm^3^/g	Mean Pore Diameter, nm
MoO_2_—WO_2_	0.4	0.0011	12.5

**Figure A1:**
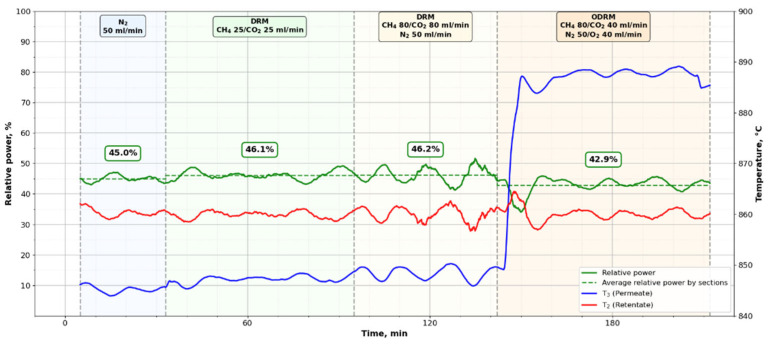
Dependence of the relative power of the installation, temperatures in the reactor volumes on volume flow rate, composition of gas mixtures and time when placing the control thermocouple in the volume of the retentate.

## Data Availability

The data presented in this study are available on request from the corresponding author.

## Appendix A

A similar experiment was conducted to confirm the independence of the temperature inversion from the placement of the control thermocouple in the membrane reactor. Figure A1 shows a change in the temperature and power of the resistor furnace when the control thermocouple is placed in the retentate volume. It can be seen that when oxygen is applied, the temperature in the retentate volume is lower than the temperature in the permeate volume and practically does not change its position over time. A description of the hypothesis explaining these changes is described in more detail in the Results (Section 3.3. Heat consumption in the DRM and ODRM processes) and Discussion.
